# Outcome of Oncology Patients Infected With Coronavirus

**DOI:** 10.1200/GO.20.00064

**Published:** 2020-03-20

**Authors:** Abdul-Rahman Jazieh, Thamer H. Alenazi, Ayman Alhejazi, Faisal Al Safi, Ashwaq Al Olayan

**Affiliations:** ^1^ Department of Oncology, King Abdulaziz Medical City, King Saud bin Abdulaziz University for Health Sciences, King Abdullah International Medical Research Center, Riyadh, Saudi Arabia

## Abstract

**PURPOSE:**

This study investigated the features of oncology patients with confirmed Middle East respiratory syndrome (MERS) at the Ministry of National Guard Health Affairs-Riyadh during the outbreak of June 2015 to determine the clinical course and outcome of affected patients.

**METHODS:**

The patients’ demographic information, cancer history, treatment pattern, information about MERS-coronavirus (CoV) infection, history of travel, clinical symptoms, test results, and outcome were collected and analyzed as part of a quality improvement project to improve the care and safety of our patients. Only patients with confirmed infection were included.

**RESULTS:**

A total of 19 patients were identified, with a median age of 66 years (range, 16-88 years), and 12 patients (63%) were males. The most common underlying disease was hematologic malignancies (47.4%), followed by colorectal cancer (21%) and lung cancer (15.8%). Hypertension and diabetes mellitus were the most common comorbidities (57.9% and 52.6%, respectively). Infection was diagnosed by nasopharyngeal swab in all patients. All patients contracted the infection during their hospitalization for other reasons. Sixteen patients (80%) were admitted to the intensive care unit; 13 patients (81%) had acute respiratory distress syndrome, 11 were intubated (68.75%), 9 had acute renal injury (56.25%), and 3 required dialysis (18.75%). Only 3 patients (15.8%) with early-stage cancers survived. Patients with hematologic malignancies and advanced solid tumors had a 100% case fatality rate. The majority of the causes of death were due to multi-organ failure and septic shock.

**CONCLUSION:**

MERS-CoV infection resulted in a high case fatality rate in patients with malignancy. Therefore, it is critical to implement effective primary preventive measures to avoid exposure of patients with cancer to the virus.

## INTRODUCTION

Since the isolation of the novel coronavirus (CoV), Middle East respiratory syndrome (MERS)-CoV, in Saudi Arabia in 2012,^1^ multiple outbreaks of this virus have occurred inside and outside of the Kingdom, including reports of infection from at least 27 countries.^2^ The MERS-CoV outbreak occurred in Riyadh in June 2015 and led to the closure of the Ministry of National Guards Health Affairs Hospital.^3^ The outbreak affected many patients and health care workers, and required a systematic approach to successfully contain the outbreak and resume full organizational services.^3,4^

Patients with cancer are usually susceptible to infectious diseases, and it is known that infections are a leading cause of death among this patient population. There are multiple causes for this vulnerability of patients with cancer related mainly to 3 factors: the underlying disease, cancer-directed treatment, and underlying comorbidities. The underlying malignancy may result in a weakened immune system due to the negative effect on immune cells, affecting their quantity or quality, as is the case in leukemia or lymphoma. Cancer therapy itself, whether it is surgery, radiotherapy, or chemotherapy, disturbs the immune system in various ways and results in an increased risk of infection. Patients with cancer are likely to have other comorbidities, whether due to age or exposure to cancer risk factors, such as smoking, obesity, and other lifestyle choices. Chronic diseases and comorbidities were identified previously as risk factors for MERS-CoV mortality.^5,6^ All these factors lead to the predisposition of patients with cancer to a high risk of acquiring infectious diseases with a worse outcome than patients without cancer.^7^

In 2015, our oncology patients were affected by the MERS-CoV outbreak and, sadly, many of them died. The outcome of oncology patients affected by MERS-CoV infection was not reported independently from other chronic diseases. In this article, we describe the characteristics and outcome of all oncology patients infected with the virus at our institution during that outbreak.

**Context**

**Key Objective**
To describe the outcome of oncology patients with confirmed MERS-CoV infection in 2015 outbreak in Saudi Arabia.
**Knowledge Generated**
During the outbreak, 19 oncology patients had confirmed infection with the virus. Only three patient (15.8%) survived the illness. This case fatality rate (CFR) is more than double the CFR of nononcology patients. The CFR reached 100% in patients with advanced cancer and hematological malignancies.
**Relevance**
Extra precautions should be taken to protect patients with cancer from exposure to infectious pathogens during outbreaks. Primary prevention such as vaccine is direly needed.

## METHODS

### Study Design

We performed a retrospective review of the medical records of eligible patients to retrieve the following variables: patients’ demographics, cancer history, treatment pattern, information about MERS-CoV infection, history of travel, clinical symptoms, test results, and survival status. All oncology patients with a laboratory-confirmed MERS-CoV infection between June 1, 2015, and September 30, 2015, were included in the analysis.

### Statistical Plan and Data Management

Descriptive statistics were used to analyze demographic and disease characteristics, presentation, treatment, use of health care resources, and survival status. Overall survival (OS) was determined from the onset of symptoms to the date of death from any cause or loss to follow-up. Patients who were still alive at the time of the analysis were censored at the date of last contact. The Kaplan-Meier method was used to estimate OS and 95% CIs for the median time of OS.

Inferential analysis was performed by applying univariable analysis to examine the association between survival status and patient demographic and clinical characteristics. The χ^2^ test was used to compare proportions of nominal categorical variables, and the *t* test was used for continuous variables. *P* values < .05 were considered significant.

## RESULTS

A total of 130 patients were infected with MERS-CoV at our hospital; 43 were health care workers.^4^ Nineteen patients with a confirmed cancer diagnosis were identified and are included in this report. Patients’ characteristics are described in Table 1. The majority of patients were males, with median age of 66 years; approximately half of the patients had a hematologic malignancy. The majority of solid tumor malignancies were at an advanced stage, and 79% of the patients had other comorbidities. Almost one third of the patients were receiving active treatment, and 90% had active disease at the time of infection (Table 1). Dyspnea was the most common symptom, followed by fever and cough. GI symptoms, such as diarrhea and vomiting, were the main symptoms outside the respiratory system (Table 2).

**TABLE 1:**
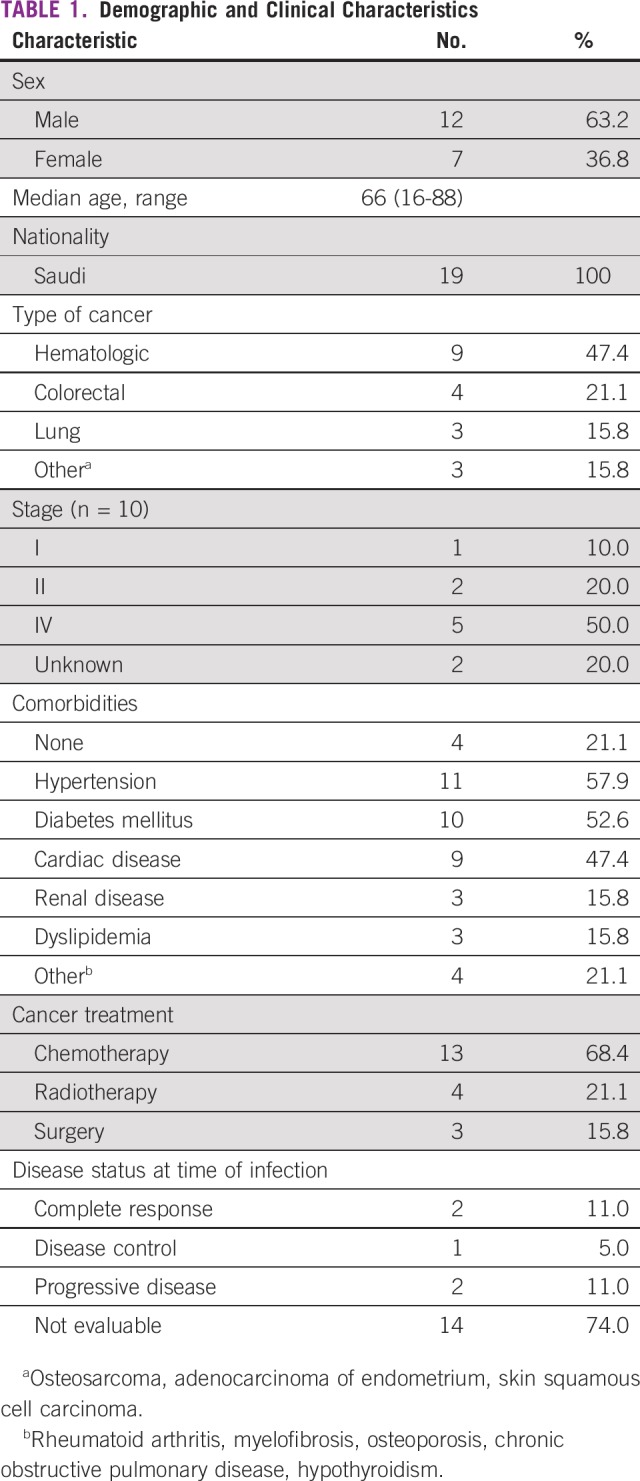
**Demographic and Clinical Characteristics**

**TABLE 2:**
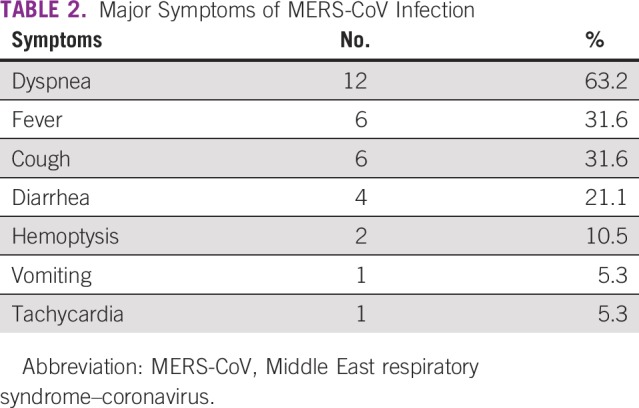
Major Symptoms of MERS-CoV Infection

Sixteen patients (80%) were admitted to the intensive care unit (ICU); 13 patients (81%) had acute respiratory distress syndrome, 11 were intubated (68.75%), 9 had acute renal injury (56.25%), and 3 required dialysis (18.75%; Table 3). After comparing patients undergoing cancer treatment versus those with nonactive treatment, no significant differences were identified between the 2 groups with respect to ICU admission or the patients’ outcomes. Sixteen patients died as a result of their disease, with multi-organ failure and sepsis being the most common causes of death (Table 4). The median time from the onset of respiratory symptoms to ICU admission was 14 days, and median survival from the onset of symptoms was 26 days. Only 3 patients survived.

**TABLE 3:**
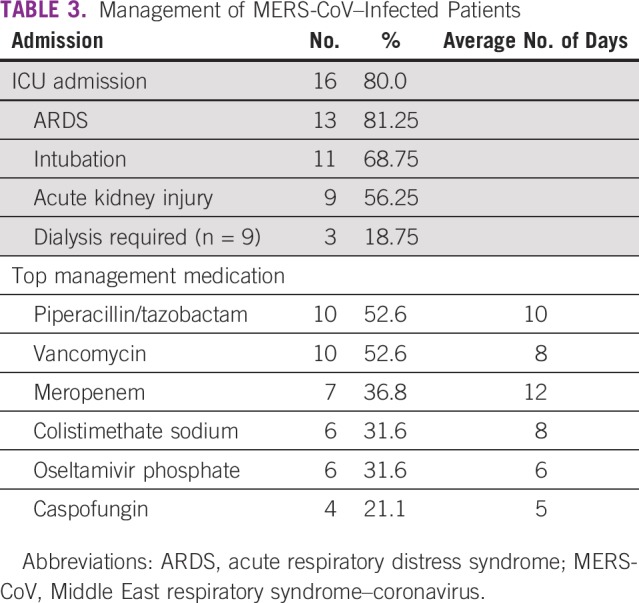
Management of MERS-CoV–Infected Patients

**TABLE 4:**
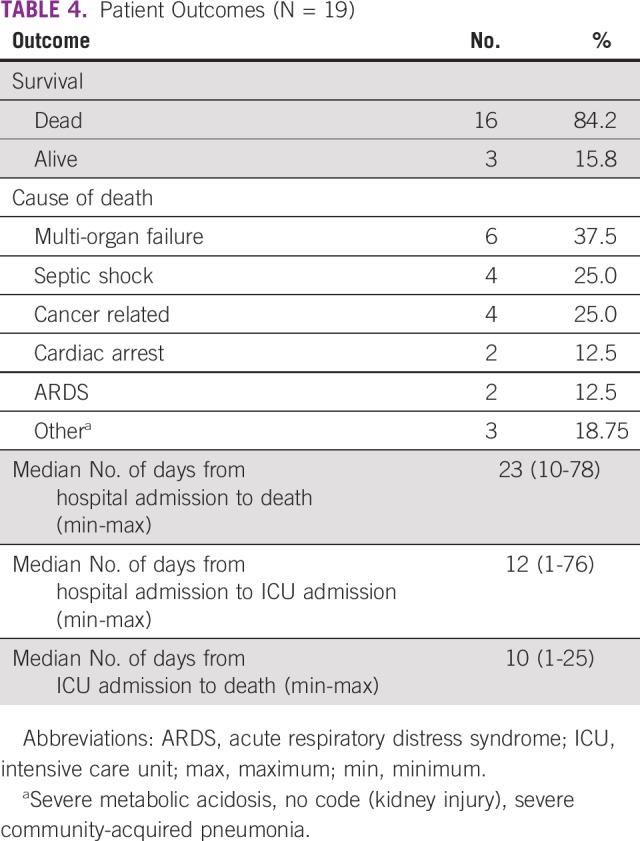
Patient Outcomes (N = 19)

Univariable analysis showed significant association between survival status and stage of cancer (*P* = .001). Late stage and hematologic malignancies were predominant factors in patients who died; active disease was significantly associated with a high fatality rate (*P* = .018; Table 5).

**TABLE 5:**
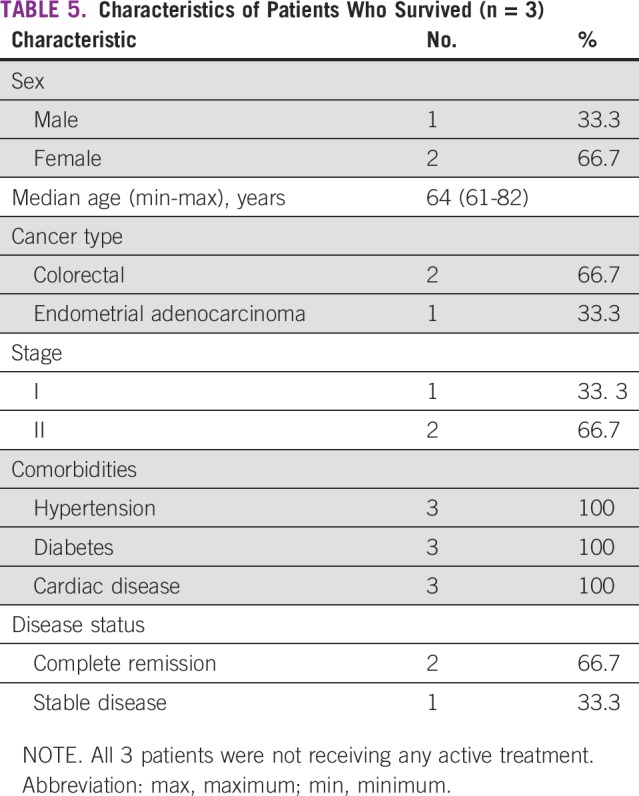
**Characteristics of Patients Who Survived (n = 3)**

## DISCUSSION

Our study revealed a high fatality rate (84%) for patients with cancer infected with MERS-CoV, reaching 100% in patients with hematologic malignancies and advanced-stage solid tumors. This is more than double the risk compared with the death rate among other patients (39%) at our hospital or what was reported in the literature (in the range of 35%).^4,8^ Although there is no reported mortality rate in only oncology patients, a Korean study revealed a death rate of 64% in patients with chronic comorbidities, including cancer.^9^ This high fatality rate can be explained by the nature of the underlying disease and other comorbidities.^10,11^ The significance of our findings dictates the need to implement extra precaution measures to ensure that these vulnerable patients are not exposed to the virus.

An important factor to note in the patient population studied is that less than one third of the patients initially presented with fever. This fraction is considered small when compared with previously reported studies conducted in Saudi Arabia between 2012 and 2013 (98%),^11^ in Al-Hasa (87%),^12^ in Makkah (63%),^13^ and in Riyadh (61%).^14^ Most of these studies reported 0%-8% malignant disease as one of the comorbidities in the study populations. This finding identifies that fever is a not a reliable symptom for the suspicion of MERS-CoV infection in patients with cancer.

A previous predictor of age > 65 years in the previous MERS-CoV outbreak from 2012-2014^11^ was not found to be a significant variable in our study population. Among patients with cancer, advanced-stage and hematologic malignancies appear to be associated with a 100% case fatality rate, because all the survivors had early-stage solid tumors.

The limitation of our study is the small number of patients and the retrospective nature of the study design. Our study does not account for any asymptomatic undiagnosed infection that the patient may have recovered from or any patients who died without a confirmed diagnosis.

It is prudent to ensure that all possible precautionary measures be implemented to protect oncology patients from being exposed to MERS-CoV; developing additional protective measures, such as a vaccine, is important to prevent infection in this vulnerable population.^12^ Developing effective antiviral treatment will help in saving the lives of affected patients.

MERS-CoV is associated with an extremely high fatality rate among patients with cancer. Fever may not be a reliable indicator of MERS-CoV infection in this population. Better preventive and therapeutic measures are urgently needed.^10,11,14^
